# Local Control of Excitation-Contraction Coupling in Human Embryonic Stem Cell-Derived Cardiomyocytes

**DOI:** 10.1371/journal.pone.0005407

**Published:** 2009-04-30

**Authors:** Wei-Zhong Zhu, Luis F. Santana, Michael A. Laflamme

**Affiliations:** 1 Department of Pathology, University of Washington, Seattle, Washington, United States of America; 2 Institute for Stem Cell and Regenerative Medicine, University of Washington, Seattle, Washington, United States of America; 3 Department of Physiology & Biophysics, University of Washington, Seattle, Washington, United States of America; University of Cincinnati, United States of America

## Abstract

We investigated the mechanisms of excitation-contraction (EC) coupling in human embryonic stem cell-derived cardiomyocytes (hESC-CMs) and fetal ventricular myocytes (hFVMs) using patch-clamp electrophysiology and confocal microscopy. We tested the hypothesis that Ca^2+^ influx via voltage-gated L-type Ca^2+^ channels activates Ca^2+^ release from the sarcoplasmic reticulum (SR) via a local control mechanism in hESC-CMs and hFVMs. Field-stimulated, whole-cell [Ca^2+^]_i_ transients in hESC-CMs required Ca^2+^ entry through L-type Ca^2+^ channels, as evidenced by the elimination of such transients by either removal of extracellular Ca^2+^ or treatment with diltiazem, an L-type channel inhibitor. Ca^2+^ release from the SR also contributes to the [Ca^2+^]_i_ transient in these cells, as evidenced by studies with drugs interfering with either SR Ca^2+^ release (i.e. ryanodine and caffeine) or reuptake (i.e. thapsigargin and cyclopiazonic acid). As in adult ventricular myocytes, membrane depolarization evoked large L-type Ca^2+^ currents (*I*_Ca_) and corresponding whole-cell [Ca^2+^]_i_ transients in hESC-CMs and hFVMs, and the amplitude of both *I*_Ca_ and the [Ca^2+^]_i_ transients were finely graded by the magnitude of the depolarization. hESC-CMs exhibit a decreasing EC coupling gain with depolarization to more positive test potentials, “tail” [Ca^2+^]_i_ transients upon repolarization from extremely positive test potentials, and co-localized ryanodine and sarcolemmal L-type Ca^2+^ channels, all findings that are consistent with the local control hypothesis. Finally, we recorded Ca^2+^ sparks in hESC-CMs and hFVMs. Collectively, these data support a model in which tight, local control of SR Ca^2+^ release by the *I*_Ca_ during EC coupling develops early in human cardiomyocytes.

## Introduction

Human embryonic stem cells (hESCs) can be induced to differentiate *in vitro* into cardiomyocytes (hESC-CMs). These cells express expected cardiac markers and exhibit spontaneous action potentials (APs), [Ca^2+^]_i_ transients, and contractile activity. At present, however, the mechanisms underlying excitation-contraction (EC) coupling in hESC-CMs are incompletely understood. Addressing this issue is critical for two fundamental reasons. *First*, hESC-CMs represent a unique model system in which to study the development of EC coupling in early human myocardium. *Second*, because of their tremendous expandability and unquestioned cardiac potential, hESC-CMs have considerable promise for eventual application in cell-based cardiac repair (for review, see refs [1], [2]). However, because hESC-CMs should be well-matched to host adult human ventricular myocardium to optimize host-graft electromechanical integration and minimize the risk of arrhythmias, the development of any cell therapies based on hESC-CMs must be preceded by a thorough investigation of the biophysical properties of these cells.

Unlike in hESC-CMs, the mechanisms underlying EC coupling in mammalian adult ventricular myocytes have been the subject of intense investigation for many years and so are comparatively well-understood (for review, see refs [3], [4]). Studies with adult cardiomyocytes have led to the formulation of the local control model of EC coupling [5]–[9]. In this model, brief openings of voltage-gated L-type Ca^2+^ channels during the AP allow a small amount of Ca^2+^ to enter the cardiac cytoplasm. This Ca^2+^ influx causes a local [Ca^2+^]_i_ increase that rapidly (<1 ms) activates nearby sarcoplasmic reticulum (SR) Ca^2+^ release channels (i.e. ryanodine receptors, RyRs) by the mechanism of Ca^2+^-induced Ca^2+^ release (CICR) [10]. The simultaneous activation of a small number of RyRs allows Ca^2+^ stored in the lumen of the SR to flow into the cardiomyocyte cytoplasm causing a local increase in [Ca^2+^]_i_. These local Ca^2+^ release events, termed ‘Ca^2+^ sparks’, are considered the elementary Ca^2+^ release events of EC coupling[6]. Ca^2+^ sparks can occur spontaneously, or they can be activated by the L-type Ca^2+^ current (*I*_Ca_) [5], [11], [12]. During the AP, activation of *I*_Ca_ synchronizes the activation of multiple Ca^2+^ sparks, which sum to produce a large, whole-cell [Ca^2+^]_i_ transient. As *I*_Ca_ inactivates, the probability of activation of Ca^2+^ sparks diminishes, thereby allowing the SR Ca^2+^ ATPase and Na^+^-Ca^2+^ exchanger to return the [Ca^2+^]_i_ to resting levels [13]. A central tenet of this model is that the amplitude of the [Ca^2+^]_i_ is graded by the amplitude of *I*_Ca_ [8].

Although the mechanisms underlying EC coupling in hESC-CMs are incompletely understood, in principle one can envision *four* potential mechanistic models for the development of a global, whole-cell [Ca^2+^]_i_ transient during an AP in these cells. *Model 1* involves a mechanism similar to that of turtle [14], frog [15], and dogfish [16] ventricular myocytes as well as primary embryonic murine cardiomyocytes [17], in which [Ca^2+^]_i_ transients result solely from Ca^2+^ influx via *I*_Ca_ during the AP. In *model 2*, Ca^2+^ influx via L-type Ca^2+^ channels activates SR Ca^2+^ release via CICR during an AP. However, in this model, the strength of the functional coupling between L-type Ca^2+^ channels and RyRs could be weak or variable [18]. In *model 3*, global [Ca^2+^]_i_ transient are produced by spontaneous release from intracellular stores, without the activation of L-type Ca^2+^ channels. This model has been reported to hold for EC coupling in primary murine embryonic cardiomyocytes and cardiomyocytes from murine ESCs [19]–[22]. Finally, *model 4* is similar to the one described above for adult ventricular myocytes, which involves tight, local coupling between Ca^2+^ influx and SR Ca^2+^ release during EC coupling.

In this study, we examined the mechanisms of EC coupling in hESC-CMs, as well as in ∼100 day old human fetal ventricular myocytes (hFVMs), which serve as a useful comparison cell type of known age. Using a variety of techniques including fluorescent Ca^2+^ imaging, voltage-clamp studies, and confocal immunofluorescence microscopy, we demonstrate that EC-coupling in both cell types involves Ca^2+^ influx via dihydropyridine-sensitive, voltage-gated L-type Ca^2+^ channels, which results in SR Ca^2+^ release via a tight, local mechanism akin to that exhibited by mature ventricular cardiomyocytes (i.e. *model 4* above).

## Materials and Methods

### Differentiation of hESC-CMs

For all experiments, H7 hESCs [23] were differentiated into cardiomyocytes using our recently reported directed cardiac differentiation protocol [24]. In brief, hESCs were expanded in the undifferentiated state on Matrigel (BD Biosciences, San Jose, CA) coated substrates using mouse embryonic fibroblast conditioned medium (MEF-CM) [25]. Prior to induction of cardiogenesis, hESCs were enzymatically dispersed, replated onto Matrigel-coated surfaces in a high-density monolayer culture, and then maintained for an additional 6 days in MEF-CM. To induce cardiac differentiation, MEF-CM is replaced by RPMI-B27 medium (Invitrogen, Carlsbad, CA) supplemented with the following cytokines: 100 ng/ml human recombinant activin A (R&D Systems, Minneapolis, MN) for 24 hours, followed by 10 ng/ml human recombinant bone morphogenenetic protein-4 (BMP-4, R&D Systems) for 4 days. This medium is then exchanged for RPMI-B27 without supplementary cytokines on every second day for an additional 10 days. Widespread spontaneous beating activity is typically observed by 9–12 days following induction with activin A. On day 14 post-induction, cells are enzymatically dispersed (with dispase) and re-plated onto polyethylenimine- and gelatin-coated glass coverslips for calcium imaging, electrophysiological recordings, or immunofluorescence 3–7 days later. We routinely immunostained comparably prepared cultures and, consistent with our prior report describing this method [24], found the majority of resultant cells to be comprised of cardiomyocytes (59±8% positive for the striated muscle marker sarcomeric actin, data not shown).

### Dissociation of human fetal ventricular myocytes

Human fetal hearts (90–110 days gestational age) were obtained from the University of Washington Birth Defects Research Laboratory under a waiver from the University's Institutional Review Board (IRB) for Human Subjects. The IRB determined that this work, which involved anonymous human biological materials received from this depository, is not considered human subjects research (IRB Determination # 08-0062-N). The NIH-funded Birth Defects Research Laboratory tissue distribution program has been separately approved by the IRB (approval #96-1825-A13) and operates in fully compliance with all relevant state and federal laws and regulations. All donors provide written informed consent prior to donating tissues to this depository, and all donated tissues would otherwise be discarded. Ventricular myocytes were then dissociated from these fetal hearts using enzymatic methods modified from those described by Ufret-Vincenty *et al.* [26]. In brief, after transport in ice-cold DMEM, each heart was retrogradely perfused with oxygenated Ca^2+^-free Tyrode's solution (37°C, pH 7.4) for 5 minutes, followed by a switch to a solution also containing 1% collagenase I (Worthington Biochemical, Lakewood, NJ), 0.01% protease, and 0.08 mM CaCl_2_. After approximately 10 minutes of digestion, the left ventricular tissue was removed, minced and filtered through nylon mesh. The resultant dissociated ventricular myocytes were maintained in Dulbecco's MEM at 25°C for up to 4 hours before use.

### Ca^2+^ imaging

We measured changes in [Ca^2+^]_i_ using the fluorescent Ca^2+^ indicator Fluo-4 (Molecular Probes, Eugene, OR). For experiments that involved the simultaneous measurement of electrophysiological signals and [Ca^2+^]_i_, cells were loaded with the penta-potassium salt of Fluo-4 (50 µM) through the patch pipette. For measurements of [Ca^2+^]_i_ that did not involved patch-clamping (i.e. Ca^2+^ sparks, SR Ca^2+^ load in paced and un-stimulated cells), myocytes were loaded with the membrane permeable acetoxymethyl-ester form of Fluo-4 (Fluo-4 AM) as previously described [27]. Coverslips containing the dye-loaded hESC-CMs or hFVMs were mounted in a chamber and superfused at a rate of 1 ml/min with solution A (see Table 1 below) at 25 °C.

**Table 1:** Composition of solutions used.

	Bath Solutions		Pipette Solution
	A	B	C
NaCl	140	140	-
KCl	5.4	-	-
CaCl_2_	1.8	2.0	-
MgCl_2_	1.0	1.0	-
CsCl	-	5	130
NaH_2_PO_4_	0.33	-	-
HEPES	10	10	10
Glucose	5	10	-
TTX	-	0.01	-
TEA-Cl	-	-	10
Mg-ATP	-	-	5

All values are expressed in mM.

Confocal imaging of whole-cell [Ca^2+^]_i_ and Ca^2+^ sparks was performed using a BioRad Radiance 2000 or Nikon Swept Field confocal system (Cambridge, MA, USA) coupled to a Nikon TE300 inverted microscope equipped with a Nikon 60× oil immersion lens (NA = 1.4). Images were analyzed with custom software written in IDL language (Research Systems, Boulder, CO, USA). Ca^2+^ sparks were identified using a computer algorithm similar to the one described by Cheng *et al.* [28]. The amplitude of the [Ca^2+^]_i_ transient evoked by the application of a Ca^2+^- and Na^+^-free (substituted with N-methyl-D-glucamine) solution containing 20 mM caffeine (1 s; via a picospritzer) was used as an indicator of SR Ca^2+^ content [29]. To ensure steady-state SR Ca^2+^ load, cells were subjected to a minimum of 10 preconditioning pulses (1 Hz) before caffeine was applied. Calibration of fluorescence signals was performed using the ‘pseudo-ratio’ equation [6].

### Patch-clamp electrophysiology

*I*_Ca_ was elicited at 1 Hz and recorded using an Axopatch 200B patch-clamp amplifier (Axon Instruments, Union City, CA) operated in voltage-clamp mode. During *I*_Ca_ recordings, cells were superfused with solution B (see Table 1 below) at 25° C. The pipette solution used in these experiments was solution C (also in Table 1). To inactivate the fast sodium current, cells were depolarized via a 500 ms duration “ramp” from a holding potential of −70 mV to −50 mV and then held at the latter potential for an additional 200 msec. To activate *I*_Ca_, cells were next depolarized with a 200 ms duration step from −50 mV to any of range of voltages from −40 to +100 mV. For some experiments, [Ca^2+^]_i_ transients were simultaneously recorded either during the depolarization step or after repolarization to a −70 mV holding potential, using the techniques detailed in the preceding section. The whole-cell capacitance was determined using the charging time (‘RC’) constant that resulted from 5 mV depolarizing pulses from a holding potential of −70 mV. The capacitance of hESC-CMs and hFVMs measured 24.6±3.3 pF (n = 15) and 20.3±4.6 pF (n = 6), respectively. The series resistance compensation circuitry of the Axopatch 200B was used in all voltage-clamp experiments to compensate for about 60% of the series resistance. Signals were digitized and stored on a computer running the pCLAMP 8 software suite (Axon Instruments), and analysis of electrophysiological records was performed using the CLAMPFIT module of pCLAMP 8. *I*_Ca_ was normalized to cell capacitance.

### Field-stimulation

Field stimulation was performed via two platinum wires (0.5 cm separation) placed at the bottom of a customized perfusion chamber. An IonOptix Myopacer (IonOptix Corp, Milton, MA, USA) stimulator was used to deliver square voltage pulses (4 ms duration) with amplitude of 1.5× threshold at a frequency of 1 Hz.

### Immunocytochemistry

Immunostaining was performed as previously described [24], [30], using antibodies directed against the RyR2 channel (mouse monoclonal antibody, Affinity Bioreagents, Rockford, IL, used at a 1∶1000 dilution) and Cav1.2, the α_1c_ subunit of the L-type Ca channel (rabbit polyclonal antibody, Sigma, used at a 1∶500 dilution). In brief, cells were fixed with 2% paraformaldehyde, permeabilized with the addition of 0.1% Triton X-100 in phosphate-buffered saline (PBS) for 10 minutes, quenched in an isotonic solution of 50 mM glycine in diluted PBS for 10 minutes, and then blocked with 1.5% normal goat serum in PBS at 4° C overnight. The aforementioned primary antibodies were then applied serially (each overnight at 4° C, also in 1.5% normal goat serum in PBS), followed by detection with either an Alexa-488-conjugated goat anti-mouse or Alexa-594-conjugated goat anti-rabbit secondary antibody (Molecular Probes, Eugene, OR). Images were acquired using the Nikon Swept Field confocal system described above with a 100× (1.49 NA) lens. Alexa-488 and Alexa-594 were excited with the 488 nm and 561 nm laser lines of this system. Fluorescence emission signals from these indicators were collected sequentially and separated using appropriate filter sets. Images were collected at 0.25 µm intervals in the z-axis. Co-localization analysis and volumetric reconstructions of confocal three-dimensional images stacks were performed using Elements software (Nikon).

### Statistics

All data groups were submitted to a D'Agostino & Pearson omnibus test to determine whether they had a normal (i.e. bell-shaped) distribution. Parametric data are presented as mean±standard error of the mean (SEM). Non-parametric data are presented as median and range. Two-sample comparisons were made using a Mann-Whitney or Student's t-test. A *p* value less than 0.05 was considered significant. Asterisks (*) used in the figures indicate a significant difference between groups.

## Results

### Ca^2+^ influx via L-type Ca^2+^ channels is required for evoking whole-cell [Ca^2+^]_i_ transients in hESC-CMs

**Figure 1:**
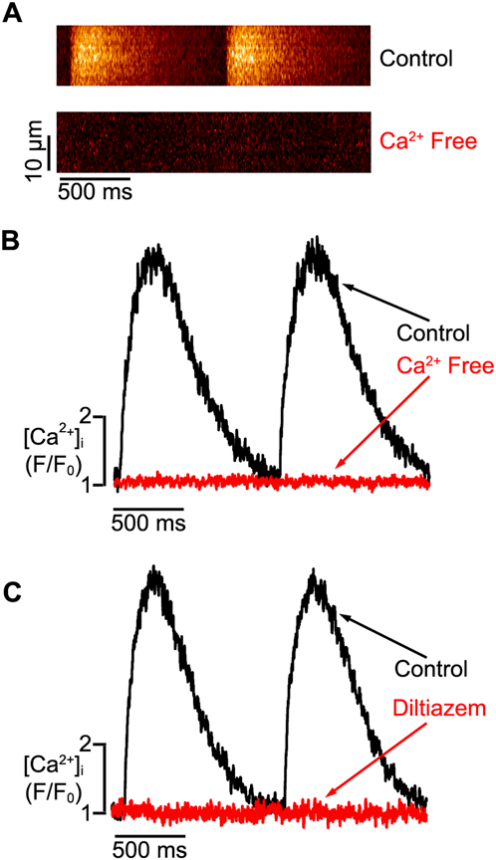
External Ca^2+^ influx via L-type Ca^2+^ channels is required for EC coupling in hESC-CMs. A. Confocal line-scan images from a representative, field-stimulated hESC-CM under control conditions (i.e. 1.8 mM external Ca^2+^, upper image) and after the application of a Ca^2+^-free solution (lower). B. Corresponding [Ca^2+^]_i_ transients under control (black trace) and Ca^2+^-free (red) conditions. C. [Ca^2+^]_i_ transients before (black) and after the application of the L-type Ca^2+^ channel blocker diltiazem (10 µM, red).

We investigated whether Ca^2+^ influx was required for the development of a global [Ca^2+^]_i_ transient during EC coupling in hESC-CMs. APs were evoked via field stimulation (1 Hz). [Ca^2+^]_i_ was recorded in cells loaded with the fluorescent Ca^2+^ indicator fluo-4 using confocal microscopy (see **Methods and Materials** section above for details). Under control conditions (i.e. 1.8 mM external Ca^2+^), APs evoked large, cell-wide [Ca^2+^]_i_ transients in hESC-CMs (Figure 1). The average amplitude of these [Ca^2+^]_i_ was 4.6±0.4 F/F_0_ (n = 19 cells). [Ca^2+^]_i_ rose in hESC-CMs after activation of the AP: the time-to-peak of the evoked [Ca^2+^]_i_ transient was 150±25 ms. Analysis of the decaying phase of the [Ca^2+^]_i_ transient revealed the time it took to decay to 50% amplitude of its amplitude (T_50_) was 245±45 ms. Note that the time-to-peak and T_50_ of the [Ca^2+^]_i_ transients in hESC-CMs are similar to those reported for [Ca^2+^]_i_ transients in adult human ventricular myocytes [31]. Removal of external Ca^2+^ eliminated AP-evoked [Ca^2+^]_i_ transients in hESC-CMs, demonstrating a requirement for Ca^2+^ influx during EC coupling in these cells (Figure 1A and 1B).

We next tested the hypothesis that Ca^2+^ influx via L-type Ca^2+^ channels is required for activation of the [Ca^2+^]_i_ transient during an AP in hESC-CMs (Figure 1C). To do this, [Ca^2+^]_i_ transients were recorded in hESC-CMs before and after the application of the L-type Ca^2+^ blocker diltiazem (10 µM, n = 9 cells). Consistent with our hypothesis, diltiazem (10 µM) eliminated the [Ca^2+^]_i_ transient in these cells (Figure 1C). Together with the external Ca^2+^ experiments described above, these findings indicate that activation of L-type Ca^2+^ channels during an AP allows Ca^2+^ influx thus inducing a global increase in [Ca^2+^]_i_ in hECM-CMs. Thus, these data are consistent with *models 1, 2, and 4* discussed in the **Introduction** section above.

### SR Ca^2+^ release amplifies Ca^2+^ influx during EC coupling in hESC-CMs

**Figure 2:**
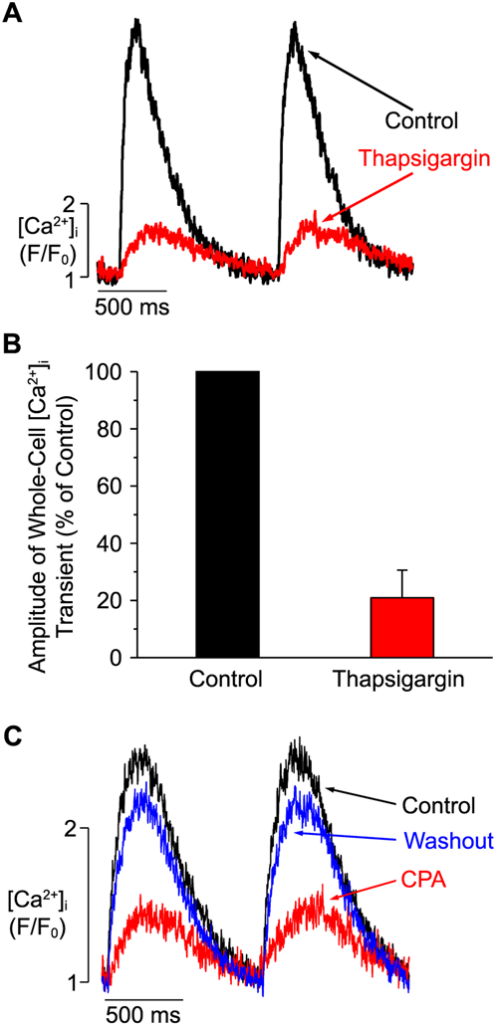
Thapsigargin decreases [Ca^2+^]_i_ transients in hESC-CMs. A. Time-course of field-stimulated [Ca^2+^]_i_ transients in a representative hESC-CM under control conditions (black trace) and after exposure to thapsigargin (1 µM, red). B. Bar graph describing the percentage change in the peak of the [Ca^2+^]_i_ transient before (control, black) and after (red) exposure to thapsigargin in hESC-CMs. C. Time-course of field-stimulated [Ca^2+^]_i_ transients in a representative hESC-CM under control conditions (black), during exposure to 10 µM cyclopiazonic acid (CPA, red), and following washout of CPA (blue).

We examined the role of SR Ca^2+^ release during EC coupling in hESC-CMs by recording [Ca^2+^]_i_ transients in these cells before and after the application of the irreversible SR Ca^2+^ATPase (SERCA) inhibitor thapsigargin (1 µM) (Figure 2A and B). Thapsigargin treatment decreased the amplitude of the evoked [Ca^2+^]_i_ transient to 21±10% of control (n = 7; *p*<0.05). We next repeated this experiment using the reversible SERCA inhibitor cyclopiazonic acid (CPA, 10 µM). Figure 2C shows AP-evoked [Ca^2+^]_i_ transients from a representative hESC-CM under control conditions, during CPA treatment, and after the SERCA pump inhibitor was washed out. CPA decreased [Ca^2+^]_i_ transient amplitude to 30±10% (n = 7; *p*<0.01), but this effect was partially reversed following drug washout (to 57±9% of baseline, n = 7; p<0.01). Taken collectively with the data in Figure 1 (see above), these findings indicate that SR Ca^2+^ release amplifies the Ca^2+^ influx via L-type Ca^2+^ channels and is a major contributor of Ca^2+^ during the evoked global [Ca^2+^]_i_ transient during EC coupling.

### Graded activation of I_Ca_ and [Ca^2+^]_i_ transients by membrane potential in hESC-CMs and hFVMs

**Figure 3:**
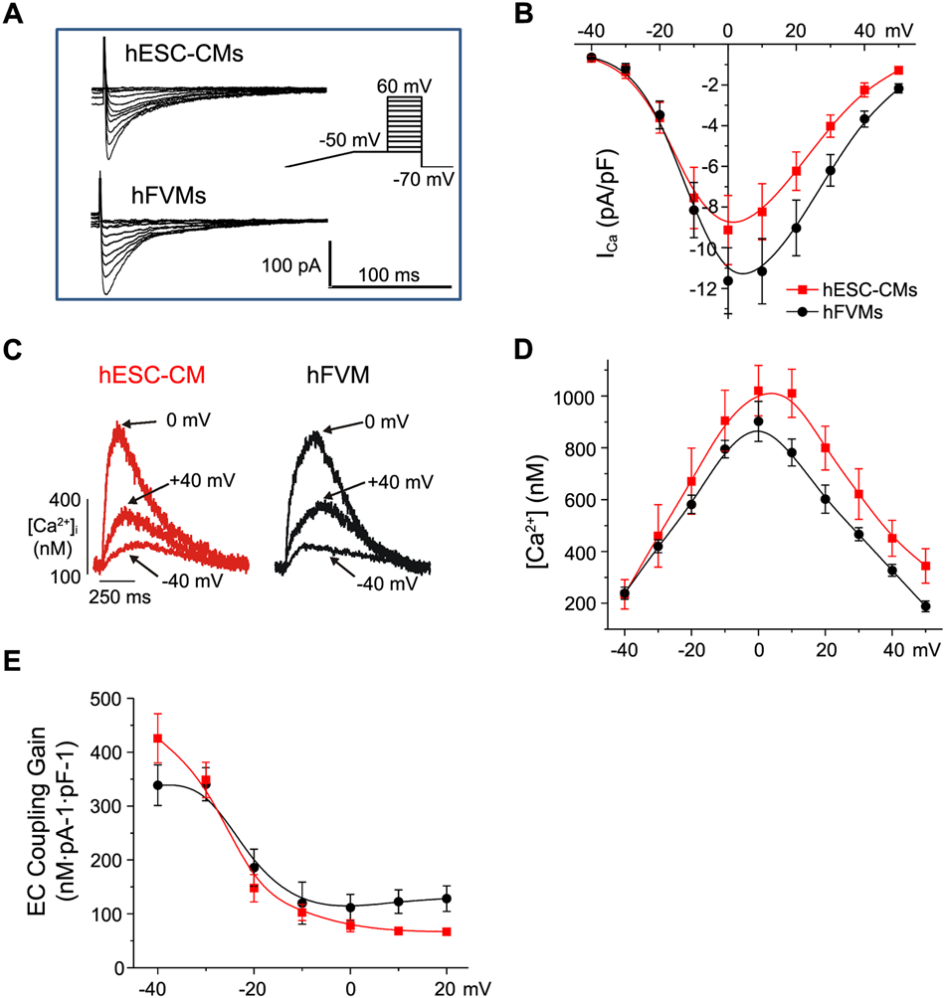
Voltage dependencies of *I*_Ca_ and [Ca^2+^]_i_ during EC coupling in hESC-CMs and hFVMs. A. *I*_Ca_ traces from typical hESC-CMs and hFVMs. *I*_Ca_ was evoked elicited using the voltage protocol depicted in the inset to the right. In brief, cells were slowly depolarized from the holding potential of −70 mV to −50 mV, where they were held to inactivate the Na^+^ current. Cells were then depolarized from −50 mV to test potentials ranging from −40 to +60 mV for 200 ms. B. Current-voltage relationships of *I*_Ca_ in hESC-CMs (red) and hFVMs (black). C. [Ca^2+^]_i_ transients evoked by *I*_Ca_ following depolarization to −40, 0, +40 mV in representative hESC-CMs and hFVMs. D. Voltage-dependence of the amplitude of the [Ca^2+^]_i_ transient in hESC-CMs and hFVMs. E. Voltage-dependence of the EC coupling gain factor in hESC-CMs and hFVMs.

Having ruled out the possibility that Ca^2+^ influx is by itself sufficient to produce the [Ca^2+^]_i_ transients observed in hESC-CMs (i.e. *model 1* discussed in the **Introduction**), we investigated whether L-type Ca^2+^ channels activate SR Ca^2+^ release during EC coupling through a loose (i.e. *model 2* above) or a tight, local control mechanism (i.e. *model 4* above). If SR Ca^2+^ release in hESC-CMs is activated by local Ca^2+^ signals produced by closely apposed L-type Ca^2+^ channels, one would expect the amplitude of the [Ca^2+^]_i_ to be a finely graded function of the amplitude of *I*_Ca_. On the other hand, if SR Ca^2+^ release in hESC-CMs is activated by Ca^2+^ signals of variable strength (as would be expected with variations in the relative location of RyRs and L-type Ca^2+^ channels) or by spontaneous SR Ca^2+^ release alone (i.e. *model 3* above), there would be a poor correlation between the amplitude of *I*_Ca_ and the associated [Ca^2+^]_i_ transient in these cells. To determine which of these potential scenarios apply to hESC-CMs, we simultaneously recorded *I*_Ca_ and [Ca^2+^]_i_ in hESC-CMs during step depolarizations (200 ms duration) from the holding potential of −50 mV to test potentials ranging from −40 to +60 mV (Figure 3). We also obtained *I*_Ca_ and [Ca^2+^]_i_ transient recordings from hFVMs, which allowed us to compare our hESC-CM data with that from a human cardiomyocyte of a known (i.e. ∼100 days gestational age) and presumably more advanced developmental stage.

Membrane depolarization evoked large *I*_Ca_ and global [Ca^2+^]_i_ transients in hESC-CMs and hFVMs. The amplitude of *I*_Ca_ and the associated [Ca^2+^]_i_ transient varied with the magnitude of the test membrane potential in both cell types (Figure 3). Interestingly, the amplitude of *I*_Ca_ and the [Ca^2+^]_i_ transient were remarkably similar in hESC-CMs and hFVMs at all membrane potentials examined. At 0 mV, *I*_Ca_ density was 9.1±1.7 (n = 7 cells) and 11.6±1.6 (n = 6 cells; *p* = 0.067) pA/pF in hESC-CMs and hFVMs, respectively. The amplitude of the [Ca^2+^]_i_ transient at the same potential was 1097±97 and 902±77 nM (*p* = 0.15) in hESC-CMs and hFVMs, respectively.

Moreover, as previously reported in adult ventricular myocytes [32], [33], the voltage dependencies of *I*_Ca_ (Figure 3B) and the [Ca^2+^]_i_ transients (Figure 3C) were bell-shaped, gradually increasing in amplitude as the cell was depolarized from −40 to 0 mV. Depolarization to more positive potentials (>0 mV) evoked progressively smaller *I*_Ca_ and [Ca^2+^]_i_ transients, likely due to a reduction in the driving force for Ca^2+^ influx. To quantify changes in the sensitivity of the SR to the trigger I_Ca_, we calculated the EC coupling “gain” (defined as the ratio of the [Ca^2+^]_i_ transient amplitude to the *I*_Ca_ current density [12], [34]) elicited with each voltage step. One of the predictions of the local control hypothesis (i.e. *model 4*) is that this gain factor will be high at negative potentials (e.g. −40 mV, where *I*_Ca_ is small, but the driving force for Ca^2+^ entry via single L-type Ca^2+^ channels is large) but will decrease with depolarization to increasingly positive test potentials (e.g. 0 mV, where *I*_Ca_ amplitude is maximal, but the amplitude of unitary L-type Ca^2+^ current is comparatively small). This prediction holds true in mammalian adult ventricular myocytes [5], [12], [34], and we found that it also applies to hESC-CMs and hFVMs (Figure 3E).

**Figure 4:**
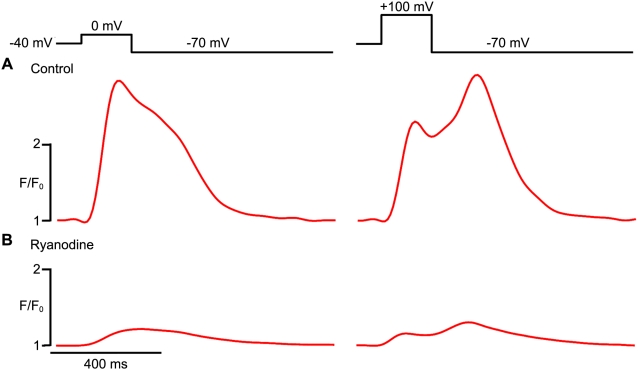
Tail [Ca^2+^]_i_ transients in hESC-CMs. A. [Ca^2+^]_i_ traces from a representative hESC-CMs during a 200 ms step depolarization from −40 mV to a test potential of 0 mV (left) or +100 mV (right), followed by a repolarization to −70 mV. Note the large tail [Ca^2+^]_i_ transient evoked by repolarization from +100 mV in the record to the right. B. Corresponding [Ca^2+^]_i_ traces elicited by the same voltage protocols after treatment with ryanodine (10 µM).

When adult ventricular are depolarized to highly positive test potentials close to the Nernst equilibrium potential for Ca^2+^ (∼+120 mV), L-type Ca^2+^ channels open, but the Ca^2+^ influx through these channels is low due to a weak driving force. However, upon repolarization from such extremely positive test potentials (e.g. repolarization from +100 mV to −70 mV), there is a rapid increase in driving force, and a “tail” *I*_Ca_ is evoked. Because L-type Ca^2+^ channels deactivate rapidly (τ of deactivation<1 ms), these tail *I*_Ca_ currents are short-lived. In adult ventricular myocytes, tail *I*_Ca_ currents can also induce [Ca^2+^]_i_ transients, a phenomenon that has been attributed to CICR initiated by the Ca^2+^ influx during repolarization [32], [35], [36]. The exhibition of tail [Ca^2+^]_i_ transients is considered strong evidence for the local control hypothesis, as the extremely brief tail *I*_Ca_ is thought to allow only a small amount of Ca^2+^ influx and so is only able to activate RyRs within a tight, local domain. The presence of tail [Ca^2+^]_i_ transients also distinguishes cardiac-type EC coupling from that in skeletal muscle, which operates on a charge-coupled mechanism and so repolarization does not elicit SR Ca^2+^ release [37]. We found that hESC-CMs exhibited robust tail [Ca^2+^]_i_ transients in response to repolarization to −70 mV following a voltage step to +100 mV (Figure 4A). Of note, both these tail [Ca^2+^]_i_ transients and [Ca^2+^] transients elicited during a voltage step to peak I_Ca_ (i.e. a test potential of 0 mV) were largely eliminated by treatment with ryanodine (10 µM), an effect that indicates a significant contribution by release from RyR-gated SR Ca^2+^ stores (Figure 4B). In particular, ryanodine reduced the amplitude of tail [Ca^2+^]_i_ transients (evoked by repolarization from +100 mV to −70 mV) from a baseline of 945±37 nM to 250±10 nM, and it reduced the amplitude of [Ca^2+^] transients (evoked by a depolarizing step to 0 mV) from 853±52 nM to 269±8 nM (n = 6 cells per condition).

Collectively, these data indicate that Ca^2+^ release from the SR is graded by the amplitude of *I*_Ca_, likely via local Ca^2+^ signaling between closely apposed L-type Ca^2+^ channels and RyRs in hESC-CMs (i.e. *model 4*).

### Co-localization of ryanodine receptors and L-type Ca^2+^ channels in hESC-CMs

**Figure 5:**
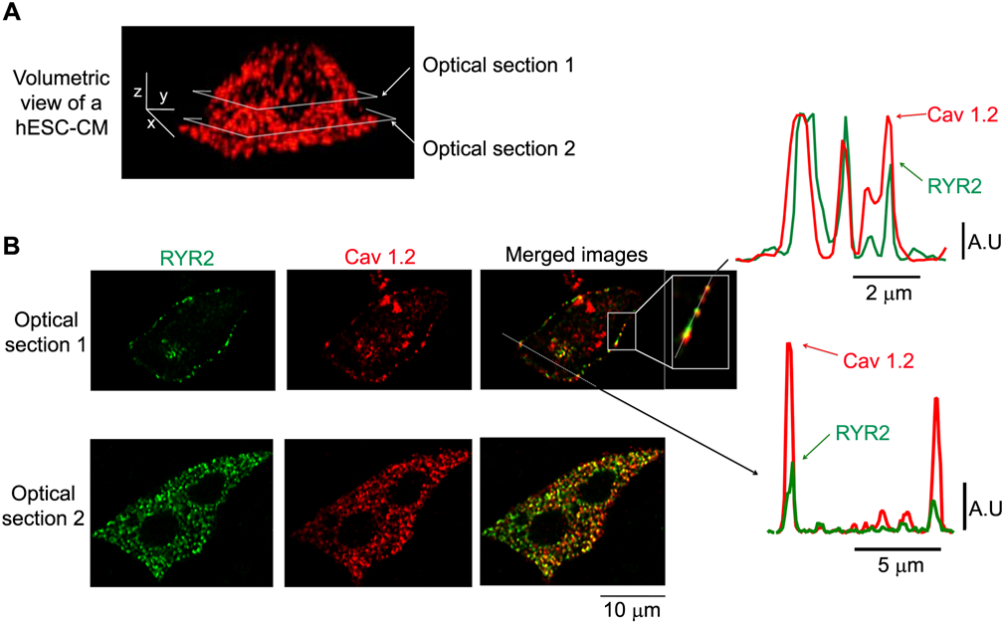
Immunofluorescent co-localization of RyR2 and L-type Ca^2+^ channels in hESC-CMs. A. Volumetric view of a stack of confocal anti-Cav1.2 images from a representative hESC-CM. Images were collected every 0.25 µm in the z-axis. The scale bars in the image indicate 5, 2, and 5 µm along the x, y, and z-axes, respectively. The horizontal grey boxes show the location of the two optical sections depicted in panel B. B. Two-dimensional anti-RyR2 (green, left), anti-L-type Ca^2+^ channels (anti-Cav1.2, red, center), and merged images, corresponding to optical sections 1 and 2 from panel A. Note that the merged image from optical section 1 includes two dotted lines, one that runs along the cell membrane (magnified in the inset) and one that traverses the cell. The corresponding plots to the right indicate the two-channel fluorescent intensity along each of the aforementioned lines.

Another testable prediction of the local control model of EC coupling is that L-type Ca^2+^ channels (Cav1.2) and ryanodine receptors (RyRs) are co-localized within specific domains near the sarcolemma. To test this hypothesis in hESC-CMs, we examined the subcellular distribution of type 2 RyRs (RyR2s) and L-type Ca^2+^ channels in hESC-CMs using confocal immunofluorescence approaches. As illustrated by Figure 5A, we observed punctate immunoreactivity for L-type Ca^2+^ channels throughout the sarcolemma of hESC-CMs. Interestingly, L-type Ca^2+^ channels (red) and RyR2s (green) were co-localized within small clusters near the surface of the cell (Figure 5B). The diameter at 50% amplitude of L-type Ca^2+^ channels and RyR2 clusters was 1.2±0.1 µm (n = 55) and 1.0±0.1 µm (n = 41), respectively. We performed quantitative co-localization analysis of L-type Ca^2+^ and RyR2-associated fluorescence on a pixel-by-pixel basis. The statistical significance of the co-localization of these two proteins is supported by Mander's overlap and Pearson correlation values of +0.88 and +0.83, respectively. These data indicate that L-type Ca^2+^ channels are co-localized with RyR2s within small sarcolemmal clusters in hESC-CMs.

### Spontaneous Ca^2+^ sparks in hESC-CM and hFVMs

**Figure 6:**
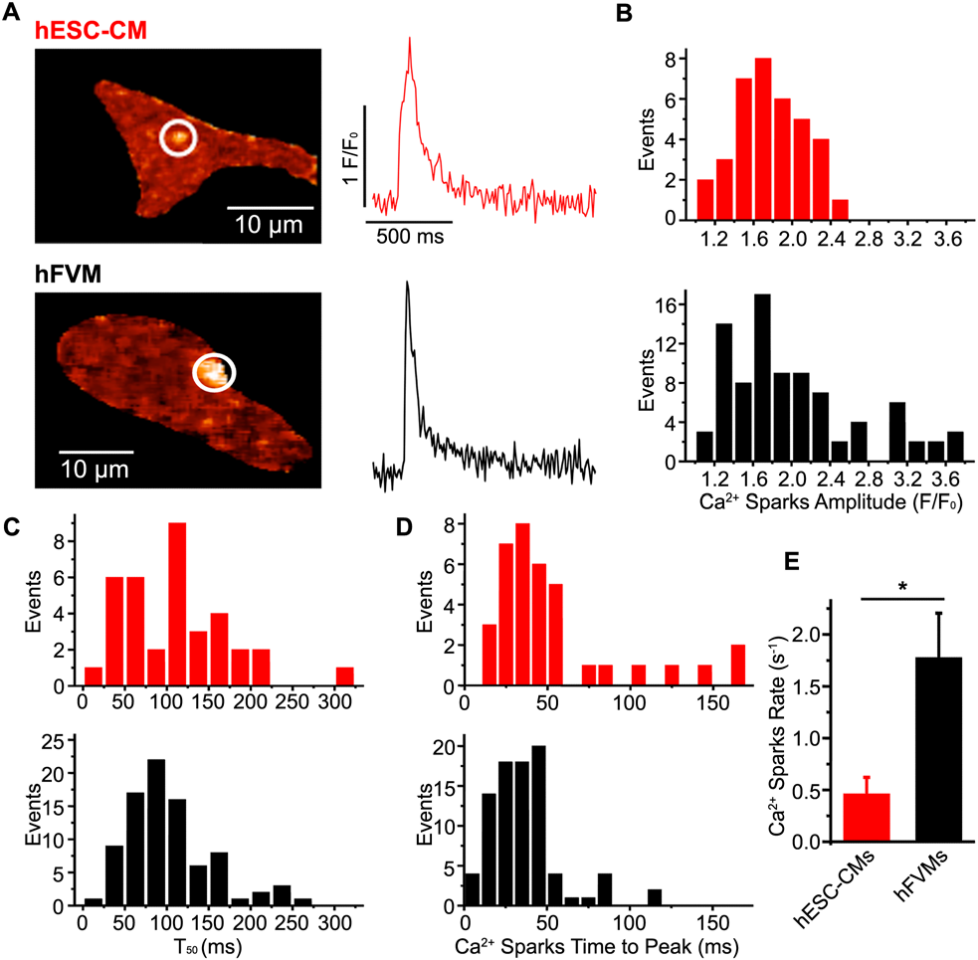
Ca^2+^ sparks in hESC-CMs and hFVMs. A. Confocal images of Ca^2+^ sparks in typical hESC-CMs and hFVMs. The traces to the right of each image show the time-course of [Ca^2+^]_i_ in each Ca^2+^ spark site (red trace: hESC-CM, black trace: hFVM). Histograms of the amplitude (B), decay time to 50% amplitude (T_50_) (C), and time-to-peak (D) for Ca^2+^ sparks in hESC-CMs (red bars, upper) and hFVMs (black bars, lower). E. Bar graph indicating the rate of Ca^2+^ spark occurrence in hESC-CMs and hFVMs. * *p*<0.05.

To better understand the mechanisms underlying Ca^2+^ release from the SR of hESC-CMs and hFVMs, we asked whether these cell types exhibit Ca^2+^ sparks, the elementary release events during EC-coupling. In fact, spontaneous Ca^2+^ sparks were routinely observed in both hESC-CMs and hFVMs. Representative sparks and associated parameters from both cell types are depicted in Figure 6, and a summary of our comparative analysis of Ca^2+^ sparks in these cells is presented in Table 2. The width at 50% peak amplitude and T_50_ were similar in both cell types (*p*>0.05, n = 36 in hESC-CMs, n = 86 in hFVMs). Interestingly, however, the Ca^2+^ spark rate (defined as the number of Ca^2+^ sparks per sec per cell) was nearly four-fold higher in hFVMs (n = 7) than in hESC-CMs (n = 10; *p*<0.01). Ca^2+^ spark amplitude and time-to-peak were also greater in hFVMs (n = 86 events) than in hESC-CMs (n = 36 events, p<0.05).

**Table 2:** Ca^2+^ spark parameters in hESC-derived and fetal ventricular cardiomyocytes.

Parameter	hESC-CMs (n = 10 cells)	hFVMs (n = 7 cells)
Frequency (Hz)	Mean = 0.45±0.17**	1.76±0.44
Amplitude (F/F_0_)	Median = 1.7*	Median = 2.0
	Range = 1.1–3.8	Range = 1.2–2.45
	(n = 36 events)	(n = 86 events)
τ_decay_ (ms)	Median = 96	Median = 85
	Range = 18–312	Range = 24–264
Time-to-peak (ms)	Median = 38*	Median = 28
	Range = 10–160	Range = 9–112
Width at 50% peak amplitude (mm)	Mean = 1.9±0.1	Mean = 1.9±0.6

** P<0.01.

* P<0.05 hESC-CMs vs. hFVMs.

**Figure 7:**
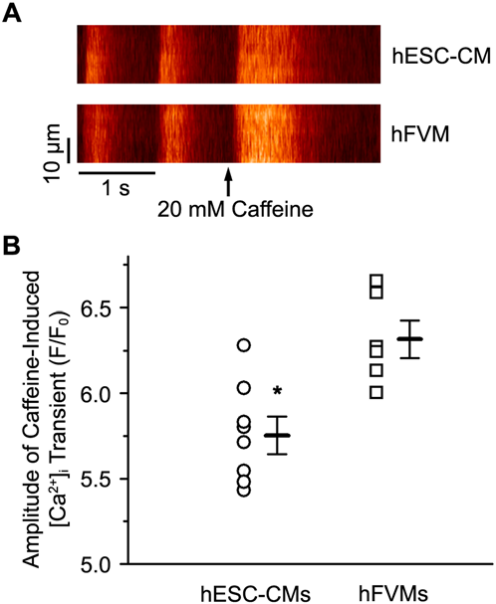
Higher SR Ca^2+^ load in hFVMs than in hESC-CMs. A. Line-scan images showing AP-evoked and caffeine-induced [Ca^2+^]_i_ transients in hESC-CMs (upper image) and hFVMs (lower image). Delivery of the 20 mM caffeine is indicated by the arrows. B. Scatter plot of the amplitude of the caffeine-induced [Ca^2+^]_i_ transient in hFVMs and hESC-CMs. The horizontal bars in the plot show the mean±SEM of the caffeine-induced [Ca^2+^]_i_ transient in each experimental group. * *p*<0.05.

Finally, because SR Ca^2+^ load modulates Ca^2+^ spark activity [38], we also examined this parameter in hESM-CMs and hFVMs, using the amplitude of a caffeine-induced [Ca^2+^]_i_ transient as an indicator of SR Ca^2+^ load (Figure 7). To ensure steady-state SR Ca^2+^ loading, caffeine (20 mM) was applied after conditioning each cell with a train of ten APs. We found that the amplitude of the caffeine-induced [Ca^2+^]_i_ transient was higher in hFVMs (6.3±0.3 F/F_0_, n = 6) than in hESC-CMs (5.8±0.3 F/F_0_, n = 8, *p* = 0.004), suggesting that steady-state SR Ca^2+^ load is greater in hFVMs than in hESC-CMs.

## Discussion

In adult ventricular myocytes, activation of SR Ca^2+^ release during the AP occurs at special structures named Ca^2+^ release units (CRUs) in which L-type Ca^2+^ channels are in close proximity (about 10–12 nm) to RyRs in the junctional SR [39]. The cytosolic volume in this junctional space is small. Brief openings (<1 ms) of L-type Ca^2+^ channels in these regions of the cell allow enough Ca^2+^ influx to produce a local, junctional increase in [Ca^2+^]_i_ (10–100 µM) to activate nearby RyRs via CICR, resulting in Ca^2+^ sparks [40]–[42]. The local control model of EC coupling holds that, under physiological conditions, the activation of any given CRU does not activate nearby release units because RyRs are relatively insensitive to Ca^2+^ and [Ca^2+^]_i_ does not reach high enough levels in neighboring sites to induce their activation. This prevents the regenerative, all-or-none [Ca^2+^]_i_ transient that would be predicted by a global control mechanism. Instead, the amplitude of the [Ca^2+^]_i_ transient is produced by the temporal and spatial summation of Ca^2+^ sparks activated by *I*_Ca_ during the AP. The amplitude of the [Ca^2+^]_i_ transient in adult ventricular myocytes is graded by the amplitude of *I*_Ca_, which determines the probability of Ca^2+^ spark recruitment during an AP.

Although hESC-CMs are known to exhibit [Ca^2+^]_i_ transients and express key Ca^2+^ handling proteins, including SERCA2b, the Na^+^/Ca^2+^ exchanger, and RyR2, there has been considerable controversy as to the fundamental mechanisms of excitation-contraction coupling in these cells [43]–[46]. For example, Dolnikov *et al* [47] concluded that essentially all of the [Ca^2+^]_i_ transient rise observed in hESC-CMs results from trans-sarcolemmal entry via calcium channels and that these cells have little or no release from internal Ca^2+^stores. By contrast, Satin *et al* [48] recently reported that hESC-CMs do exhibit caffeine- and ryanodine-sensitive SR Ca^2+^stores, even at early stages (e.g. as soon as 2 days following the appearance of spontaneous beating activity). A third group, Liu et al. [45], also found evidence for functional SR Ca^2+^stores in hESC-CMs, albeit only in a minority of cardiomyocytes. Of note, all three groups largely relied on hESC-CMs generated using the “historical” method of embryoid body differentiation (i.e. the formation of spontaneously differentiating three-dimensional aggregates in high fetal calf serum (FCS)). The present study is the first to examine intracellular Ca^2+^ signaling in hESC-CMs resulting from our recently reported guided differentiation protocol (involving serial activin A and BMP-4 under serum-free, monolayer culture conditions). This method results in preparations of substantially higher cardiac purity (typically >60% cardiomyocytes, versus <1% cardiomyocytes via embryoid body differentiation), greatly facilitating biophysical studies with hESC-CMs, but it could in principle result in cardiomyocytes with a different functional phenotype. For example, FCS is known to retard cardiac differentiation [49], so its elimination in our protocol could conceivable promote a greater degree of maturation.

Here, we report that that hESC-CMs produce Ca^2+^ sparks and exhibit robust *I*_Ca_ and [Ca^2+^]_i_ transients. *I*_Ca_ density in hESC-CM is similar to that of hFVM as well as adult human, mouse, and rat ventricular myocytes [31], [50], [51]. Note that the capacitance — an indicator of cell surface area — of hESC-CMs and hFVMs is ∼20–25 pF, which is roughly 5–10-fold smaller than that of adult ventricular myocytes (which typically range between 100–200 pF [52]). Collectively, these data suggest that *I*_Ca_ density is established early in the differentiation of ventricular cardiomyocytes and that, during development, myocyte hypertrophy is accompanied by a concomitant increase in L-type Ca^2+^ channel expression (presumably Cav1.2) that leads to a relatively stable *I*_Ca_ density throughout development.

The findings in our study, which is the first to directly examine the relationship between *I*_Ca_ and [Ca^2+^]_i_ transients in hESC-CMs, are consistent with a model in which activation of SR Ca^2+^ release is tightly regulated by Ca^2+^ signals via L-type Ca^2+^ channels during EC coupling. The data supporting this model are compelling. *First*, Ca^2+^ influx via functional L-type Ca^2+^ channels is necessary for EC coupling in hESC-CMs. *Second*, Ca^2+^ influx via L-type Ca^2+^ channels activate SR Ca^2+^ release in these cells, thereby producing a global increase in [Ca^2+^]_i_ that triggers contraction. *Third*, SR Ca^2+^ release is not all-or-none in hESC-CMs. Instead, as in adult ventricular myocytes [32], [33], SR Ca^2+^ release is finely graded by the amplitude of the L-type Ca^2+^ current during EC coupling. *Fourth*, large [Ca^2+^]_i_ transients were evoked by tail *I*_Ca_ during repolarization from +100 mV to −70 mV. *Fifth*, EC coupling gain is larger at negative potentials where *I*_Ca_ is small (i.e. −40 mV), but the driving force for Ca^2+^ influx is large, than at more positive potentials in which *I*_Ca_ is maximal (i.e. 0 mV), but the driving force for Ca^2+^ entry is lower. *Sixth*, RyR2 and L-type Ca^2+^ channels co-localize in specific regions of the sarcolemma of hESC-CMs. Collectively, these findings strongly indicate that the establishment of tight, local control of EC coupling is an early event in the development of human cardiomyocytes. Note that this conclusion distinguishes hESC-CMs from developing cardiomyocytes in model species, including mice [17], [20]–[22], rats [53], [54], and rabbits [55], [56], in which such adult-like mechanisms of EC coupling have not generally been observed. Future studies to further investigate these species differences are clearly warranted (and ideally should include larger, slower-rated animal models, which we speculate may more closely approximate developing human cardiomyocytes).

As with the magnitude and kinetics of the *I*_Ca_, we found that the amplitude of the evoked [Ca^2+^]_i_ transient in hESC-CMs was similar to that of hFVMs as well as adult human [57], mouse [27], and rat ventricular myocytes [6]. Our data suggest that a combination of Ca^2+^ influx and SR Ca^2+^ release (presumably via Ca^2+^ sparks, but see below) translates into a global [Ca^2+^]_i_ transient during the physiological AP in hESC-CMs. Interestingly, the relative contribution of *I*_Ca_ (≈20%) and the SR (≈80%) to the [Ca^2+^]_i_ transient in these cells is similar to that of adult human ventricular myocytes [57]. These observations suggest that the amplitude and relative contributions of Ca^2+^ influx and release from intracellular release are established early in cardiac biogenesis and remain stable throughout development. Multiple factors come into play in the generation of the [Ca^2+^]_i_ transient in ventricular myocytes, including the amount of Ca^2+^ influx, SR Ca^2+^ release, the buffering capacity of the cell as well as the rate and capacity of Ca^2+^ extrusion, and SR Ca^2+^ re-sequestration mechanisms. For example, while the absolute values for cytosolic buffering capacity, accessible volume, Ca^2+^ influx and SR Ca^2+^ release likely differ between hESC-CMs, hFVMs, and adult ventricular myocytes, it is clear their relative proportions lead to comparable AP-evoked changes in [Ca^2+^]_i_ in these cells.

hESC-CMs and hFVMs also showed Ca^2+^ sparks, and their sparks were similar in amplitude, duration, and spatial spread to those reported in adult cardiomyocytes [6], [58]. Our data suggests that, just as *I*_Ca_ appears fairly early in the development of ESC-CMs [59], [60], hESC-CMs at an early stage of maturation possess functional CRUs capable of producing adult-like Ca^2+^ sparks. We infer that this early development of CRUs is also likely to be true in human heart development *in situ*. Ca^2+^ spark rate was lower in hESC-CMs than in hFVMs presumably because SR Ca^2+^ load was higher in hESC-CMs than in hFVMs these cells [38]. The latter data raise an interesting conundrum: How can hFVMs have larger SR Ca^2+^ load than hESC-CMs, but Ca^2+^ sparks and whole-cell [Ca^2+^]_i_ of similar amplitude? Our data do not provide an answer to this difficult question. One intriguing possibility is that the number of RyRs underlying a Ca^2+^ spark differs between hESC-CMs and hFVMs. Thus, while the total Ca^2+^ flux through each RyR channel may be smaller (due to lower SR Ca^2+^ load) in hESC-CMs than in hFVMs, a larger number of these channels per CRU in hESC-CMs results in Ca^2+^ sparks of similar amplitude in both cell types. If correct, this implies that, while hESC-CMs have functional CRUs capable of producing Ca^2+^ sparks, the development of these units is likely incomplete. Future studies should examine the mechanisms underlying the development of CRUs and Ca^2+^ sparks in hESC-CMs in more detail.

The Kamp [61] and Mummery [46] groups have reported that hESC-CMs are electrophysiologically diverse and include myocytes with distinct pacemaker/nodal-like and “working” (atrial and ventricular chamber- like) AP properties. While it would be of considerable interest to correlate hESC-CM cardiac subtype with the mechanisms of EC-coupling, this is unfortunately technically challenging and so was not performed in the present study. However, in separate current-clamp studies using the same cell preparations, we found that ∼70% (11 of 16) of cells exhibited a “working”, ventricular-like AP phenotype, with the balance showing nodal-like APs (data not shown). Thus, we expect that the vast majority of recordings in this study were obtained with ventricular-like cardiomyocytes. Interestingly, unlike our AP data, we found the EC-coupling parameters in these cells to be highly homogenous (i.e. with a unimodal distribution), inviting speculation that EC-coupling mechanisms may be similar across cardiac subtypes at this developmental stage. We plan to revisit this issue in more detail in future work.

To conclude, our data clearly indicate that there is a close relationship between *I*_Ca_ and [Ca^2+^]_i_ in hESC-CMs. Furthermore, our findings support the view that tight, presumably local, control of EC coupling is an early event in human cardiac development. Note that this conclusion distinguishes hESC-CMs from developing cardiomyocytes in model species, including mice [17], [20]–[22], rats [53], [54], and rabbits [55], [56], in which such adult-like mechanisms of EC coupling have not generally been observed. Finally, based on our findings, we speculate that it is total number of L-type Ca^2+^ channels and RyRs—not their density or coupling strength—that changes during the maturation of human cardiomyocytes.
